# Definition and Properties of the Libera Operator on Mixed Norm Spaces

**DOI:** 10.1155/2014/590656

**Published:** 2014-02-20

**Authors:** Miroslav Pavlovic

**Affiliations:** Faculty of Mathematics, University of Belgrade, Studentski Trg 16, P.O. Box 550, 11001 Beograd, Serbia

## Abstract

We consider the action of the operator *ℒg*(*z*) = (1 − *z*)^−1^∫_*z*_^1^‍*f*(*ζ*)*dζ* on a class of “mixed norm” spaces of analytic functions on the unit disk, *X* = *H*_*α*,*ν*_^*p*,*q*^, defined by the requirement *g* ∈ *X*⇔*r* ↦ (1 − *r*)^*α*^ *M*_*p*_(*r*, *g* ^(*ν*)^) ∈ *L* ^*q*^([0,1], *dr*/(1 − *r*)), where 1 ≤ *p* ≤ ∞, 0 < *q* ≤ ∞, *α* > 0, and *ν* is a nonnegative integer. This class contains Besov spaces, weighted Bergman spaces, Dirichlet type spaces, Hardy-Sobolev spaces, and so forth. The expression *ℒg* need not be defined for *g* analytic in the unit disk, even for *g* ∈ *X*. A sufficient, but not necessary, condition is that $\sum_{n=0}^{\infty }‍|\hat{g}(n)|/(n+1)<\infty$. We identify the indices *p*, *q*, *α*, and *ν* for which 1°*ℒ* is well defined on *X*, 2°*ℒ* acts from *X* to *X*, 3° the implication $g\in X\Rightarrow \sum_{n=0}^{\infty }‍|\hat{g}(n)|/(n+1)<\infty$ holds. Assertion 2° extends some known results, due to Siskakis and others, and contains some new ones. As an application of 3° we have a generalization of Bernstein's theorem on absolute convergence of power series that belong to a Hölder class.

## 1. Introduction and Definitions

Let *H*(*𝔻*) denote the class of all functions holomorphic in the unit disk *𝔻* of the complex plane. In [1], Libera introduced the operator

(1) $$\begin{array}{c} g\left(z\right)⟼\frac{2}{z}\int_{0}^{z}g\left(\zeta \right)d\zeta \end{array}$$

and showed its importance in the theory of univalent functions. In particular, it was shown in [1] that this operator transforms the class of star-like functions into itself. Since then many papers were published devoted to this aspect of the Libera operator. The “generalized” Libera operator

(2) $$\begin{array}{c} \Lambda_{a}g\left(z\right)=\frac{1}{a-z}\int_{z}^{a}g\left(\zeta \right)d\zeta ,\;\text{where}\;\;\left|a\right|\leq 1, \end{array}$$

was introduced and studied from the functional analytic point of view by Siskakis in [2, 3], then in [4–6], and other papers (see [7] for further references). If |*a* | <1, then Λ_*a*_ is defined on *H*(*𝔻*), and, on classical spaces such as Hardy, Bergman, and Besov, has almost the same linear topological properties as the integration operator *g*(*z*) ↦ ∫_0_^*z*^ *g*(*ζ*)*dζ*, and therefore is not so interesting from the functional analytic point of view (cf. [8]). So we can assume that |*a* | = 1. In fact we can and will assume that *a* = 1, so

(3) $$\begin{array}{c} \Lambda_{1}g\left(z\right)=\frac{1}{1-z}\int_{z}^{1}g\left(\zeta \right)d\zeta , \end{array}$$

whenever the integral is somehow defined. This definition of Λ_1_ requires further explanation because the integral need not be defined for *g* ∈ *H*(*𝔻*) (e.g., *g*(*z*) = 1/(1 − *z*)).

Endowed with the topology of uniform convergence on compact subsets of *𝔻*, the class *H*(*𝔻*) becomes a complete locally convex space. The dual of *H*(*𝔻*) is equal to $H(\bar{𝔻})$, where $g\in H(\bar{𝔻})$ means that *g* is holomorphic in a neighborhood of $\bar{𝔻}$ (depending on *g*). The duality pairing is given by

(4) $$\begin{array}{c} \langle f,g\rangle =\sum_{n=0}^{\infty }\hat{f}\left(n\right)\hat{g}\left(n\right), \end{array}$$

where $f(z)=\sum_{n=0}^{\infty }\hat{f}(n)z^{n}\in H(𝔻)$ and $g(z)=\sum_{n=0}^{\infty }\hat{g}(n)z^{n}\in H(\bar{𝔻})$ (see, e.g., [9]). Clearly, Λ_1_ is well defined on $H(\bar{𝔻})$, and it is easy to check that it maps $H(\bar{𝔻})$ into $H(\bar{𝔻})$, and

(5) Λ1g(z)=∑n=0∞(∑k=n∞g^(k)k+1)zn=∫01g(t+(1−t)z)dt.

The last integral is obtained from (3) by integration over the straight line joining *z* and 1.

**Definition 1**

We use the symbol $\bar{ℒ}$ to denote the operator $\Lambda_{1}:H(\bar{𝔻})\mapsto H(\bar{𝔻})$.

**Definition 2**

We denote by *ℒ* the operator *ℒg*(*z*) = ∫_0_^1^ *g*(*t* + (1 − *t*)*z*)*dt* whenever the integral converges uniformly on compact subset of *𝔻*. “Uniform convergence” means that the limit

(6) $$\begin{array}{c} \underset{x\to 1^{-}}{\lim}\int_{0}^{x}g\left(t+\left(1-t\right)z\right)dt \end{array}$$

is uniform with respect to *z*. This hypothesis guarantees that *ℒg* is an analytic function.

It is easy to verify the validity of the following.

**Proposition 3**

The dual operator ${\left(\bar{ℒ}\right)}^{*}:H(𝔻)\mapsto H(𝔻)$ coincides with the Cesàro operator, *𝒞*, defined on *H*(*𝔻*) by

(7) $$\begin{array}{c} 𝒞f\left(z\right)=\sum_{n=0}^{\infty }\left(\frac{1}{n+1}\sum_{k=0}^{n}\hat{f}\left(k\right)\right)z^{n}. \end{array}$$

Conversely, the dual of *𝒞* : *H*(*𝔻*) ↦ *H*(*𝔻*) coincides with $\bar{ℒ}$.

In [6], it was proved that *ℒ* is defined on the Bloch space and maps it into BMOA. This assertion, which improves the earlier result that *ℒ* maps Bloch space into itself (e.g., [4, 10]), was deduced from a result of Nowak [11] and Proposition 3.

However, as it can easily be seen, the operator $\bar{ℒ}$ cannot be extended to a continuous operator from *H*(*𝔻*) to *H*(*𝔻*). Moreover, $\bar{ℒ}$ cannot be extended to a continuous operator from *X* to *H*(*𝔻*), where *X* is some of common spaces, for example,

(8) X={g∈H(𝔻):    ||g||X=∫𝔻|g(z)|2(1−|z|)2  dA(z)<∞},

see [6]. (*dA* denotes the Lebesgue measure on *𝔻*). We will identify a large family of spaces that possess the same property.

In this paper we consider, in particular, the spaces listed in the following definition.

**Definition 4**

Bloch type spaces:

(9) $$\begin{array}{c} {𝔅}_{\alpha }=\left\{g\in H\left(𝔻\right):\left|g^{\prime }\left(z\right)\right|=𝒪{\left(1-\left|z\right|\right)}^{-\alpha }\right\},\;\alpha >0. \end{array}$$

Weighted Hardy spaces:

(10) $$\begin{array}{c} H_{\alpha }^{p}=\left\{g\in H\left(𝔻\right):M_{p}\left(r,g\right)=𝒪\left({\left(1-r\right)}^{-\alpha }\right)\right\},\;\alpha \geq 0, \end{array}$$

where

(11) $$\begin{array}{c} M_{p}\left(r,h\right)={\left(\frac{1}{2\pi }\int_{0}^{2\pi }{\left|h\left(re^{i\theta }\right)\right|}^{p}\;d\theta \right)}^{1/p}. \end{array}$$

Weighted Bergman spaces:

(12) Aβp={g∈H(𝔻):∫𝔻|g(z)|p(1−|z|2)β  dA(z)<∞},                     β>−1,  1≤p<∞.

Dirichlet type spaces:

(13) $$\begin{array}{c} {𝒟}_{\beta }^{p}=\left\{g\in H\left(𝔻\right):f^{\prime }\in A_{\beta }^{p}\right\}\cdot \end{array}$$

The space *𝒟*_0_^1^ is closely related to *H* ^1^ in that *H* ^1^ ⊗ *H* ^1^ = *𝒟*_0_^1^, where *X* ⊗ *Y* denotes the set of all *g* ∈ *H*(*𝔻*) which can be represented as *g* = ∑_*n*=0_^*∞*^ *h*_*n*_∗*k*_*n*_, *h*_*n*_ ∈ *X*, *k*_*n*_ ∈ *Y*, with ∑||*h*_*n*_||_*X*_||*k*_*n*_||_*Y*_ < *∞* (see [12]).

Concerning these spaces, we mention the following results.

Theorem A (see [2]). *The operator ℒ acts as a bounded operator from H* ^*p*^ *to H* ^*p*^ *if and only if *1 < *p* ≤ *∞*.

Theorem B (see [4, 10]). *ℒ acts as a bounded operator from 𝔅*_*α*_ *into 𝔅*_*α*_ *if and only if *0 < *α* < 2.

Theorem C (see [3]). *ℒ acts as a bounded operator from A* ^*p*,*β*^ *into A* ^*p*,*β*^ *if and only if p* > *β* + 2.

Theorem D (see [13]). *If* $\hat{g}(n)\geq 0$ *for all n, and* $\sum \left(\hat{g}(n)/\left(n+1\right)\right)<\infty$ *, then* *ℒg* ∈ *𝒟*_0_^1^ *if and only if*

(14) $$\begin{array}{c} \sum_{n=0}^{\infty }\frac{\hat{g}\left(n\right)\log\left(n+2\right)}{n+1}<\infty . \end{array}$$

Here “acts” means, among other things, that *ℒg* is defined on the space in the sense of Definition 2; that is,

(15) Ig(z) :=∫01g(t+(1−t)z)dt converges  uniformly  in  |z|<ρ

for all *ρ* ∈ (0,1).

Condition (15) is implied, as is easily seen, by

(16) $$\begin{array}{c} \sum_{n=0}^{\infty }\frac{\left|\hat{g}\left(n\right)\right|}{n+1}<\infty . \end{array}$$

This condition is satisfied, according to Hardy's inequality, for *g* ∈ *H* ^1^ and therefore for *g* ∈ *H* ^*p*^ ∪ *𝒟*_0_^1^, *p* ≥ 1, because *H* ^*p*^ ⊂ *H* ^1^ and *𝒟*_0_^1^ ⊂ *H* ^1^. However, (15) does not imply (16) even in the case when *ℒ* maps a space into itself. This can be seen from the following reformulation of Bernstein's theorem and Theorem 5 below.

Theorem E (Zygmund, [14], Ch. VI (3.5)). *Let *0 < *α* < 1*. Then the implication g* ∈ *H*_*α*_^*∞*^⇒*(16) holds if and only if *0 < *α* < 1/2.

**Theorem 5**

*ℒ* acts as a bounded operator from *H*_*α*_^*∞*^ into *H*_*α*_^*∞*^ if and only if 0 < *α* < 1.

(The proof is very easy, although the theorem is a special case of Theorem 11.)

What we can deduce from condition (15) is that the series

(17) $$\begin{array}{c} \sum \frac{\hat{g}\left(n\right)}{n+1}\;\;\;\;\text{is}\;\text{Abel}\;\;\text{summable}. \end{array}$$

Namely, taking *z* = 0, we see that the integral

(18) $$\begin{array}{c} I_{g}\left(0\right)=\int_{0}^{1}g\left(t\right)dt=\underset{x\to \infty }{\lim}\frac{\hat{g}\left(n\right)x^{n+1}}{n+1} \end{array}$$

exists and is finite. However, it may happen that *ℒ* maps a space into itself and that

(19) $$\begin{array}{c} \left|\hat{g}\left(n\right)\right|\geq {\left(n+1\right)}^{s}, \end{array}$$

where *s* > 0 is positive constants. For instance, as an application of Khinchin's inequality and a profound result of Kisliakov, we have the following.

**Theorem 6**

Let 1/2 ≤ *α* < 1. Then there is a function *g* ∈ *H*_*α*_^*∞*^ such that $|\hat{g}(n)|\geq c{\left(n+1\right)}^{\alpha -1/2}$ (although *ℒ* maps *H*_*α*_^*∞*^ into *H*_*α*_^*∞*^).

**Proof**

See the proof of Proposition 34, Case (1), *p* = *∞*.

On the other hand, if a space *X* satisfies (15) for all *g* ∈ *X*, this does not mean that *ℒ* maps *X* into *X* (although it is defined on *X*). Besides *H* ^1^, we have, for instance, the following.

Theorem F (see [13]). *The space 𝒟*_0_^1^ *is contained in H* ^1^ *and satisfies (16), but* *ℒ does not map 𝒟*_0_^1^ *into H* ^1^.

One of the aims of the present paper is to extend Theorems A, B, C, 5, and 6 to a large scale of “mixed norm” spaces.

**Definition 7**

We denote by *H*_*α*_^*p*,*q*^ (0 < *p*, *q* ≤ *∞*), where *α* > 0 when *q* < *∞*, and *α* ≥ 0 when *q* = *∞*, the class of those *g* ∈ *H*(*𝔻*) for which

(20) $$\begin{array}{c} \int_{0}^{1}M_{p}^{q}\left(r,f\right){\left(1-r\right)}^{\alpha q-1}dr<\infty \;\left(q<\infty \right),\\ \underset{0<r<1}{\sup}{\left(1-r\right)}^{\alpha }M_{p}\left(r,f\right)<\infty \;\left(q=\infty \right). \end{array}$$

Then letting *ν* be a nonnegative integer, we define the space

(21) $$\begin{array}{c} H_{\alpha ,\nu }^{p,q}=\left\{g\in H\left(𝔻\right):g^{\left(\nu \right)}\in H_{\alpha }^{p,q}\right\}. \end{array}$$

The (quasi) norm in *H*_*α*,*ν*_^*p*,*q*^ is given by

(22) ||g||α,νp,q  =∑j=0ν−1|g(j)(0)|       +(∫01Mpq(r,g(ν)(1−r)qα−1dr)1/q,

where ∑_*j*=0_^−1^ should be interpreted as equal to zero.

It is well known and easy to prove that these spaces are complete.

The norm (22) is not the most natural one but is convenient for technical reasons. For instance, in the case *p* = *q*, *ν* = 0, a more (but not most) natural norm is given by

(23) (∫01Mpp(r,g)(1−r)pα−1  r  dr)1/p =(12π∫𝔻|g(z)|p(1−|z|)pα−1dA(z))1/p.

This norm is equivalent to that given by (22) because of the maximum modulus principle for analytic functions.

The space *H*_*α*,*ν*_^*p*,*∞*^ = :*H*_*α*,*ν*_^*p*^ is specific in that the set, *𝒫*, of all analytic polynomials is not dense in it. The closure of *𝒫* in *H*_*α*,*ν*_^*p*^ coincides with the “little oh” space

(24) $$\begin{array}{c} h_{\alpha ,\nu }^{p}=\left\{g\in H\left(𝔻\right):M_{p}\left(r,g^{\left(\nu \right)}\right)=o{\left(1-r\right)}^{-\alpha }\left(r↑1\right)\right\}. \end{array}$$

From now on, unless specified otherwise, we suppose that

(25) $$\begin{array}{c} 1\leq p\leq \infty ,\;\;0<q\leq \infty ,\\ \alpha >0\;\text{for}\;q<\infty ,\;\;\alpha \geq 0\;\text{for}\;q=\infty . \end{array}$$

It is sometimes more convenient to work with the Besov type spaces.

**Definition 8**

Let *β* ∈ ℝ and choose any integer *ν* ≥ 0 such that *ν* − *β* > 0. The space *𝔅*_*β*_^*p*,*q*^ is defined as

(26) $$\begin{array}{c} {𝔅}_{\beta }^{p,q}=H_{\nu -\beta ,\nu }^{p,q}. \end{array}$$

If *q* = *∞*, we can assume that *ν* = *β*. It is well known that this definition is independent of *ν*; this follows immediately from Lemma A below. If *β* > 0, then *𝔅*_*β*_^*p*,*q*^ is “true” Besov spaces; if *β* < 0, then *𝔅*_*β*_^*p*,*q*^ = *H*_−*β*_^*p*,*q*^; if *β* = 0, then it is called Hardy-Bloch spaces [15]. If *q* = *∞*, then we have the space

(27) 𝔅βp={g∈H(𝔻):Mp(r,g(ν))=𝒪((1−r)β−ν)}                    (0<β<ν),

and its subspace

(28) 𝔟βp={g∈H(𝔻):Mp(r,g(ν))=o((1−r)β−ν)                     (0<β<ν)}.

*Lipschitz Spaces.* It is known that the space *𝔅*_*β*_^*p*,*q*^ (*β* > 0) coincides with the (Lipschitz) space Λ_*β*_^*p*,*q*^ consisting of those *g* in the *p*-Hardy space *H* ^*p*^ for which

(29) $$\begin{array}{c} {\left(\int_{0}^{1}{\left(\frac{{||\Delta_{t}^{\nu }g||}_{p}}{t^{\beta }}\right)}^{q}\frac{dt}{t}\right)}^{1/q}<\infty . \end{array}$$

Here Δ_*t*_^*ν*^ *g* denotes the symmetric *ν*th difference with step *t*,

(30) Δtνh(θ)=∑k=0ν(νk)(−1)ν−kh(θ+kt),         where  h(θ)=g(eiθ),

and ||·||_*p*_ denotes the norm in *H* ^*p*^. This result is essentially due to Hardy and Littlewood and Zygmund (see [16] for a simple proof of a generalized variant). We also have

(31) 𝔅βp=Λβp:=Λβp,∞={g∈Hp:||Δtng||p=𝒪(tβ)}                     (0<β<ν),𝔟βp=λβp={g∈Hp:||Δtng||p=o(tβ)(t↓0)}                  (0<β<ν).

In the case *p* = *q* = *∞* and *β* = 1, the space Λ_*β*_^*p*,*q*^ is denoted by Λ_∗_ and is called the Zygmund space. The corresponding “little” space is denoted by *λ*_∗_. These spaces were introduced by Zygmund via symmetric differences. In [17], connections of *λ*_∗_ and *λ*_∗_ with Besov spaces were established.

*Hardy-Sobolev Spaces.* Let *ν* ≥ 1 be an integer. The Hardy-Sobolev space *W*_*ν*_^*p*^ consists of those *g* ∈ *H* ^*p*^ such that *g* ^(*ν*)^ ∈ *H* ^*p*^; that is, *W*_*ν*_^*p*^ = *H*_0,*ν*_^*p*,*∞*^. It is known that

(32) $$\begin{array}{c} W_{\nu }^{p}=\left\{g\in H^{p}:\underset{0<t<1}{\sup}{||\Delta_{t}^{\nu }g||}_{p}<\infty \right\}. \end{array}$$

See, for example, [16]. In particular, *W*_1_^*∞*^ coincides with the usual Lipschitz space consisting of those *f* from the disk-algebra for which

(33) $$\begin{array}{c} \left|f\left(\zeta \right)-f\left(\eta \right)\right|\leq C\left|\zeta -\eta \right|\;\left(\left|\zeta \right|=\left|\eta \right|=1\right). \end{array}$$

There are various inclusions between members of the scale *H*_*α*,*ν*_^*p*,*q*^. Here we mention the following.

**Proposition 9**

If *α* > 0, then

- *H*_*α*,*ν*_^*p*,*q*^⫋*h*_*α*,*ν*_^*p*^⫋*H*_*α*,*ν*_^*p*^ *, for q* < *∞*,
- *H*_*α*,*ν*_^*p*,*q*^⫋*H*_*β*,*ν*_^*u*,*q*^ *, where β* = 1/*p* − 1/*u* + *α, where u* > *p, and *0 < *q* ≤ *∞*,
- *h*_*α*,*ν*_^*p*^⫋*h*_*β*,*ν*_^*u*^ *, where β* = 1/*u* − 1/*p* + *α, and where u* > *p*.

**Proof**

We can assume that *ν* = 0. Besides, we omit the proof that the inclusions are strict because we do not need this fact.

- If ∫_0_^1^ *M*_*p*_^*q*^(*r*, *g*)(1 − *r*)^*qα*−1^ *dr* < *∞*, then *M*_*p*_(*r*, *g*)(1 − *r*)^*α*^ → 0 (*r* → 1^−^) because *M*_*p*_(*r*, *g*) increases with *r*.
- It is well known that $M_{u}(r,g)\leq C{\left(1-r\right)}^{1/u-1/p}M_{p}(\sqrt{r},g)$ for *u* > *p* (see, e.g., [18, Corollary 5.1.2]).
- This follows from (b) (*q* = *∞*) and the density of *𝒫* in *h*_*α*,*ν*_^*p*^.

Let

(34) $$\begin{array}{c} \kappa_{p,\alpha ,\nu }=\nu -\alpha +1-\frac{1}{p}. \end{array}$$

We will determine the indices *p*, *q*, *α*, and *ν* for which:

1. *ℒ* acts from *H*_*α*,*ν*_^*p*,*q*^ into *H*_*α*,*ν*_^*p*,*q*^[*κ*_*p*,*α*,*ν*_ > 0];
2. *ℒ* acts from *H*_*α*,*ν*_^*p*,*q*^ to *H*(*𝔻*) but not to *H*_*α*,*ν*_^*p*,*q*^ [*κ*_*p*,*α*,*ν*_ = 0 and 1 ≤ *p* ≤ 2 and *q* ≤ 1; see Theorem 16];
3. *ℒ* acts from *H*_*α*,*ν*_^*p*,*q*^ into *H*_*α*,*ν*_^*p*,*q*^ but the implication *g* ∈ *H*_*α*,*ν*_^*p*,*q*^⇒(16) does not hold (*p* > 2 and (either *q* > 1 and *ν* − *α* ≤ −1/2, or 0 < *q* ≤ 1 and *ν* − *α* < −1/2); see Theorem 18);
4. the operator $\bar{ℒ}$ cannot be extended to a bounded operator from *H*_*α*,*ν*_^*p*,*q*^ to *H*(*𝔻*) (1°*κ*_*p*,*α*,*ν*_ < 0, 2°*κ*_*p*,*α*,*ν*_ = 0 and (*p* > 2 or *q* > 1); see Theorem 16).

Observe that, since *ν* − *α* > 1/*p* − 1 in (3), the inequality *ν* − *α* < 1/2 is equivalent to

(35) $$\begin{array}{c} \frac{1}{p}-1<\nu -\alpha <-\frac{1}{2}, \end{array}$$

which has sense because 1/*p* − 1 < −1/2 due to the condition *p* > 2.

In proving (1) we use the inequality

(36) r1/pMp(r,(ℒg)(ν)) ≤21/p(1−r)−δ−1∫r1Mp(s,g(ν))(1−s)δds,

where *δ* = *ν* − 1/*p*, which is a relatively simple consequence of the Littlewood subordination principle. A generalized version of (36) immediately gives sufficient conditions for *ℒ* to map *X* = *H*_*α*,*ν*_^*p*,*q*^ to *X*. In order to prove that these conditions are necessary we analyze membership in *X* of functions with nonnegative, nonincreasing coefficients and apply this to the Libera transform of functions with positive coefficients.

Let *f* be a function of the form

(37) $$\begin{array}{c} f\left(z\right)=\sum_{n=0}^{\infty }a_{n}z^{n},\;a_{n}\geq 0\;\;\forall n, \end{array}$$

and define *ℒf* by Definition 2. This definition is correct if and only if

(38) $$\begin{array}{c} \sum_{k=0}^{\infty }\frac{a_{k}}{k+1}<\infty , \end{array}$$

because a series with positive coefficients is Abel summable if and only if it is summable in the ordinary sense. If (38) is satisfied, then the sequence of the Taylor coefficients of *ℒf* is

(39) $$\begin{array}{c} b_{n}=\sum_{k=n}^{\infty }\frac{a_{k}}{k+1} \end{array}$$

and is therefore * nonincreasing*. In this paper we consider only the functions where *a*_*n*_ = (*n* + 1)^*γ*^/log⁡^*ε*^(*n* + 2). Discussion of the general case will appear in a separate paper.

In considering (2) and (4) we use, besides functions with positive coefficients, a deep theorem of Kolmogorov and Khinchin, while in the case of (3) we need another deep result, due to Kisliakov. By use of these theorems we can say much more about (3). Namely, if the implication *g* ∈ *H*_*α*,*ν*_^*p*,*q*^ does not hold, then there exists a function *g* ∈ *H*_*α*,*ν*_^*p*,*q*^ such that $\left|\hat{g}(n)|\geq \log^{-1}(n+2\right)$, and in some cases $|\hat{g}(n)|\geq 1$ or even $|\hat{g}(n)|\geq {\left(n+1\right)}^{\eta }$ for some *η* > 0 (see Theorem 5 and Theorem 41).

## 2. Results

Before stating our first result we give a sufficient condition for the validity of (15). This condition is not necessary (see Theorem 16).

**Proposition 10**

If *g* ∈ *H*_*α*,*ν*_^*p*,*q*^, where 0 < *p* ≤ *∞*,  0 < *q* ≤ *∞*, and

(40) $$\begin{array}{c} \nu -\alpha >\frac{1}{p}-1, \end{array}$$

then the integrals

(41) Jm(z):=∫01(1−t)mg(m)(t+(1−t)z)dt,               m≥0,  z∈𝔻

converge uniformly on compact subsets of *𝔻*, and the operator

(42) $$\begin{array}{c} ℒg\left(z\right)=\int_{0}^{1}g\left(t+\left(1-t\right)z\right)dt \end{array}$$

maps *H*_*α*,*ν*_^*p*,*q*^ to *H*(*𝔻*) and coincides with $\bar{ℒ}$ on $H(\bar{𝔻})$, and we have

(43) $$\begin{array}{c} {\left(ℒg\right)}^{\left(\nu \right)}\left(z\right)=\int_{0}^{1}{\left(1-t\right)}^{\nu }g^{\left(\nu \right)}\left(t+\left(1-t\right)z\right)dt. \end{array}$$

**Proof**

By the well-known theorem from complex analysis, it is enough to prove that *J*_0_(*z*) converges uniformly. Since *M*_*p*_(*r*, *g*) increases with *r*, the condition *g* ∈ *H*_*α*,*ν*_^*p*,*q*^ implies that

(44) $$\begin{array}{c} M_{p}\left(r,g^{\left(\nu \right)}\right)\leq C{\left(1-r\right)}^{-\alpha }. \end{array}$$

This implies, by the well-known estimate $M_{\infty }(r,h)\leq CM_{p}(\sqrt{r},h){\left(1-r\right)}^{-1/p}$, that

(45) $$\begin{array}{c} M_{\infty }\left(r,g^{\left(\nu \right)}\right)\leq C{\left(1-r\right)}^{-\alpha -1/p}. \end{array}$$

Hence, by successive integration we get *M*_*∞*_(*r*, *g*) ≤ *Cψ*(*r*), where

(46) ψ(r)=(1−r)−α−1/p+ν,   if  −1<−α−1p+ν<0   =log⁡41−r, if  −α−1p+ν=0   =1, if  −α−1p+ν>0.

It turns out that

(47) ∫01|g(t+(1−t)z)|dt ≤C∫01ψ((1−|t+(1−t)z|))dt ≤C∫01ψ((1−t−(1−t)|z|))dt =C∫01ψ((1−t)(1−|z|))dt.

It is not difficult to check that if |*z* | <*ρ* < 1, the *ψ*((1 − *t*)(1 − |*z*|)) ≤ *C*Ψ(*ρ*, *t*), where ∫_0_^1^Ψ(*ρ*, *t*)*dt* < *∞*. This implies, by Weierstrass' theorem, that *J*_0_(*z*) converges uniformly on compact subsets of *𝔻*. The rest of the proof is easy.

Our first result is as follows.

**Theorem 11**

Let *X* = *H*_*α*,*ν*_^*p*,*q*^ or *X* = *h*_*α*,*ν*_^*p*^. Then the following assertions are equivalent:

- the operator *ℒ* acts as a bounded operator from *X* into *X*;
- Condition (40) is satisfied.

Observe that condition (40) is independent of *q*.

**Remark 12**

In other words, the theorem says the following (excluding Hardy and Hardy-Sobolev spaces): let *X* = *𝔅*_*β*_^*p*,*q*^ or *X* = *𝔟*_*β*_^*p*^,  *β* ∈ ℝ. Then *ℒ* acts from *X* to *X* if and only if *β* > 1/*p* − 1, or what is the same, if and only if *β* > −1 and *p* > 1/(*β* + 1).

Theorem 11 contains some known and some new results as special cases.

**Case 1**

Theorem 11 covers the case when *α* = 0,  *q* = *∞*. In particular,

- *ℒ* maps *H* ^*p*^ into *H* ^*p*^ if and only if 1 < *p* ≤ *∞*. This is Theorem A;
- if *ν* ≥ 1, then
- *ℒ* maps Hardy-Sobolev space *W*_*ν*_^*p*^ = *H*_0,*ν*_^*p*^ into *W*_*ν*_^*p*^, for every *p* ∈ [1, *∞*], and, in particular, *ℒ* maps the ordinary Lipschitz space into itself.

This is, maybe, a new result.

**Case 2 (Theorem C)**

In particular, *ℒ* does not act as a bounded operator from *A*_*β*_^1^ into *A*_*β*_^1^, for any *β* > −1.

This is seen from Theorem 11 by taking *β* = *αp* − 1; that is, *α* = (*β* + 1)/*p*.

**Case 3**

(a) *ℒ* maps the Dirichlet space *𝒟*_*β*_^*p*^ into itself if and only if *p* > 1 + *β*/2. In particular, *ℒ* maps *𝒟*_*β*_^1^ if and only if −1 < *β* < 0.

Another case: (b) *ℒ* maps *𝒟*_*β*_^2^ into itself if and only if *β* < 2.

These facts are, maybe, new.

**Case 4**

(a) *ℒ* maps *H*_*α*_^*p*,*q*^ into itself if and only if *α* < 1 and *p* > 1/(1 − *α*).

This is seen from Theorem 11 by taking *ν* = 0. This is related to a result of [19], which, when reformulated in our notation, gives the assertion (a) under the additional condition *αq* − 1 ≥ −(1/*p*), or equivalently *qα* ≤ 1 and *p* ≤ 1/(1 − *qα*). For example, if *q* = 1 and *α* = 1/2, then this assertion says nothing because of the hypothesis *p* ≤ 2, while we still have that *ℒ* maps *H*_1/2_^*p*,1^ into itself if and only if *p* > 2.

If *α* = 0, and *ν* = 0, *q* = *∞*, then (a) says that *ℒ* maps *H* ^*p*^ into *H* ^*p*^ if and only if *p* > 1. (Compare Case 1.) In particular *ℒ* maps *H*_*α*_^*∞*^ into *H*_*α*_^*∞*^ if and only 0 ≤ *α* < 1 (this is Theorem 5). Also *ℒ* maps *h*_*α*_^*p*^ : = *h*_*α*,0_^*p*^ if and only if 0 < *α* < 1 − 1/*p*.

**Case 5**

(a) *ℒ* maps *𝔅*_*β*_^*p*,*q*^ = Λ_*β*_^*p*,*q*^ into itself for every *p*(≥1), *q*, and *β* > 0. The same holds for the little space *𝔟*_*β*_^*p*^ = *λ*_*β*_^*p*^.

This is seen from Theorem 11 (or Remark 12): the inequality *ν* + 1 > *ν* − *β* + 1/*p* holds for any *β* > 0 and *p* ∈ [1, *∞*]. In the case *q* ≥ 1, a direct proof can be found in [7]. In [6], assertion (a) (*q* ≥ 1) is proved by using the relation 𝒞* = *ℒ* and the fact that *𝒞* maps *H*_*α*_^*p*′,*q*′^ (1/*s* + 1/*s*′ = 1) into itself. (The latter was proved in [20]; a quick proof is given in [21].) What is new here is that (a) holds for *q* < 1.

(b) As a special case of (a) (*p* = *q* = *∞*) we have that *ℒ* maps the Lipschitz (=Hölder) class Λ_*β*_ = Λ_*β*_^*∞*^ into itself.

**Case 6**

*ℒ* maps *p*-Bloch type spaces *𝔅*_*α*_^*p*^ = *H*_*α*,1_^*p*^ into itself if and only if *α* + 1/*p* < 2.

The same holds for the little space *𝔟*_*α*_^*p*^ = *h*_*α*,1_^*p*^.

In particular, when *p* = *∞*, this condition reduces to 0 < *β* < 2; this is Theorem B.

**Case 7**

(a) *ℒ* maps the Hardy-Bloch space *𝔅* ^*p*,*q*^ if and only if *p* > 1.

This is, maybe, new. In the case 2 < *p* = *q* < *∞*, there exists a better result [6]: *ℒ maps 𝔅* ^*p*,*p*^ *into H* ^*p*^ (the well-known result of Littlewood and Paley states that *H* ^*p*^⫋*𝔅* ^*p*,*p*^).

**Case 8**

*ℒ* maps the Zygmund space Λ_∗_ into itself. The same holds for *λ*_∗_.

The implication (a)⇒(b) of Theorem 11 is valid because of the following two propositions.

**Proposition 13**

Let (1) *κ*_*p*,*α*,*ν*_ ≤ 0,*α* > 0, and *X* ∈ {*H*_*α*,*ν*_^*p*^, *h*_*α*,*ν*_^*p*^}, or (2) *κ*_*p*,*α*,*ν*_ < 0 and *X* = *H*_*α*,*ν*_^*p*,*q*^ (*q* < *∞*).

Then the operator $\bar{ℒ}$ cannot be extended to a bounded operator from *X* to *H*(*𝔻*).

As mentioned in Introduction (see (4), in page 4, or Theorem 16 below), the class of such spaces is larger.

**Remark 14**

In (1), the condition *α* > 0 is necessary since *H*_0,*ν*_^*p*^ is either a Hardy space (*ν* = 0) or a Hardy-Sobolev space *W*_*ν*_^*p*^(*ν* ≥ 1), which is contained in *H* ^*p*^.

**Proposition 15**

If *q* < *∞* and *κ*_*p*,*α*,*ν*_ = 0, then the function

(48) $$\begin{array}{c} f\left(z\right)=\sum_{n=0}^{\infty }\frac{z^{n}}{\log^{ɛ}\left(n+2\right)},\;where\;\max\left\{\frac{1}{q},1\right\}<ɛ\leq 1+\frac{1}{q}, \end{array}$$

belongs to *H*_*α*,*ν*_^*p*,*q*^; the function *ℒf* is well defined but *ℒf* is not in *H*_*α*,*ν*_^*p*,*q*^.

However, if *κ*_*p*,*α*,*ν*_ = 0, it may happen that *ℒ* is well defined on *H*_*α*,*ν*_^*p*,*q*^ (but, by Proposition 15, does not map the space into itself). The following theorem together with Proposition 10 characterizes those *X* = *H*_*α*,*ν*_^*p*,*q*^ such that $\bar{ℒ}$ can be extended to a bounded operator from *X* to *H*(*𝔻*).

**Theorem 16**

Let *X* = *H*_*α*,*ν*_^*p*,*q*^ (0 < *q* ≤ *∞*) or *X* = *h*_*α*,*ν*_^*p*^, and *κ*_*p*,*α*,*ν*_ ≤ 0. Then the following four conditions are equivalent:

- the operator $\bar{ℒ}$ can be extended to a bounded operator from *X* to *H*(*𝔻*);
- *ℒ* acts as a bounded operator from *X* to *H*(*𝔻*);
- If *g* ∈ *X*, then $\sum \left(\left|\hat{g}(n)\right|/\left(n+1\right)\right)<\infty$;
- 1 ≤ *p* ≤ 2, 0 < *q* ≤ 1, and *κ*_*p*,*α*,*ν*_ = 0.

A natural question arises from this theorem: under condition (d), find a quasi-Banach *X* such that *ℒ*(*H*_*α*,*ν*_^*p*,*q*^) ⊂ *X*. It turns out that we can take, for instance,

(49) X={g∈H(𝔻):∫01Mpq(r,g)            ×(1−r)qα−1log⁡−ɛlog⁡41−rdr<∞},                             ɛ>1.

**Theorem 17**

Let *ɛ* > 1, 1 ≤ *p* ≤ 2,  *κ*_*p*,*α*,*ν*_ = 0 (i.e., *α* = *ν* + 1 − 1/*p*), and *q* ≤ 1. Then *ℒ* maps *H*_*α*,*ν*_^*p*,*q*^ to *X*, where *X* is defined by (49).

This theorem can easily be deduced from (36); we will omit the proof.

There are cases when *κ*_*p*,*α*,*ν*_ > 0 (which implies that *ℒ* is well defined on *X*) but the assertion (c) of Theorem 16 does not hold.

**Theorem**

Let *X* = *H*_*α*,*ν*_^*p*,*q*^ (0 < *q* ≤ *∞*, *α* > 0) and *κ*_*p*,*α*,*ν*_ > 0. Then assertion (c) of Theorem 16 does not hold if and only if one of the following two conditions is satisfied:

1. 2 < *p* ≤ *∞*,  1 < *q* ≤ *∞*, and *ν* − *α* ≤ −1/2,
2. 2 < *p* ≤ *∞*,  0 < *q* ≤ 1, and *ν* − *α* < −1/2.

**Remark 19**

In the case of Besov type spaces, this theorem says the following: let *β* > 1/*p* − 1. The implication $g\in {𝔅}_{\beta }^{p,q}\Rightarrow \sum_{n=0}^{\infty }|\hat{g}(n)|/(n+1)<\infty$ does not hold if and only if either (1) 2 < *p* ≤ *∞*, 1 < *q* ≤ *∞*, and *β* ≤ −1/2 or (2) 2 < *p* ≤ *∞*, 0 < *q* ≤ 1, and *β* < −1/2.

Theorem 18 can be used to get a generalization of Bernstein's theorem to the case of the Besov spaces.

**Theorem 20**

Let *β* > 1/*p*. The implication

(50) $$\begin{array}{c} g\in {𝔅}_{\beta }^{p,q}⟹\sum_{n=1}^{\infty }\left|\hat{g}\left(n\right)\right|<\infty \end{array}$$

does not hold if and only if either (1) 2 < *p* ≤ *∞*, 1 < *q* ≤ *∞*, and *β* ≤ 1/2, or (2) 2 < *p* ≤ *∞*, 0 < *q* ≤ 1, and *β* < 1/2.

**Proof**

This follows from Theorem 18, the definition of Besov type spaces (Definition 8), and the equivalence *g* ∈ *𝔅*_*β*_^*p*,*q*^⇔*g*′ ∈ *H*_*ν*−*β*,*ν*−1_^*p*,*q*^.

By taking *p* = *q* = *∞* we get Bernstein's theorem.

## 3. Proof of Theorem 11

We need a variant of the Littlewood subordination principle.

Theorem G (see [22]). *If φ* : *𝔻* ↦ *𝔻 is an analytic function and g* ∈ *H* ^*p*^ *, then f*∘*φ* ∈ *H* ^*p*^ *and*

(51) $$\begin{array}{c} {||g\circ \varphi ||}_{p}\leq {\left(\frac{1+\left|\varphi \left(0\right)\right|}{1-\left|\varphi \left(0\right)\right|}\right)}^{1/p}{||g||}_{p}. \end{array}$$

Here ||*g*||_*p*_ denotes the norm of *g* in *H* ^*p*^,

(52) $$\begin{array}{c} {||g||}_{p}=\underset{0<r<1}{\sup}{\left(\frac{1}{2\pi }\int_{0}^{2\pi }{\left|g\left(re^{i\theta }\right)\right|}^{p}d\theta \right)}^{1/p}. \end{array}$$

As an application we have the following lemma.

**Lemma 21**

If *a* and *b* are positive real numbers such that *a* + *b* ≤ 1 and if *g* ∈ *H* ^*p*^,  0 < *p* ≤ *∞*, then

(53) (∫02π|g(a+beiθ)|pdθ)1/p ≤(2a+bb)1/p(∫02π|g((a+b)eiθ)|pdθ)1/p.

**Proof**

The case *p* = *∞* is easy. Let *p* < *∞*. Let *h*(*z*) = *g*((*a* + *b*)*z*),  *a*_1_ = *a*/(*a* + *b*), *b*_1_ = *b*/(*a* + *b*), and *φ*(*z*) = *a*_1_ + *b*_1_ *z*. Then

(54) ∫02π|g(a+beiθ)|pdθ =∫02π|h(a1+b1eiθ)|pdθ ≤1+a11−a1∫02π|h(eiθ)|pdθ =2a+bb∫02π|g((a+b)eiθ)|pdθ,

which was to be proved.

**Lemma 22**

If *ℒg* is well defined (see Definition 2), then

(55) r1/pMp(r,(ℒg)(ν)) ≤21/p(1−r)−(δ+1)∫r1Mp(s,g(ν))(1−r)δdr,

where *δ* = *ν* − 1/*p*.

**Remark 23**

The integral in (55) diverges if and only if *p* = 1 and *ν* = 0.

**Proof**

Let *h* = *ℒg*. We have

(56) $$\begin{array}{c} h\left(z\right)=\int_{0}^{1}g\left(t+\left(1-t\right)z\right)dt,\;z\in 𝔻, \end{array}$$

and hence

(57) $$\begin{array}{c} h^{\left(\nu \right)}\left(z\right)=\int_{0}^{1}{\left(1-t\right)}^{\nu }g^{\left(\nu \right)}\left(t+\left(1-t\right)z\right)dt. \end{array}$$

Applying the Minkowski inequality and Lemma 21 with *a* = *t*,  *b* = (1 − *t*)*r* we obtain

(58) Mp(r,h(ν)) ≤∫01(1−t)νMp(t+(1−t)r,g(ν))(2t+(1−t)r(1−t)r)1/pdt ≤21/pr−1/p∫01(1−t)ν−1/pMp(t+(1−t)r,g(ν))dt,                       1≤q<∞.

Substituting *t* + (1 − *t*)*r* = *s* and taking *ν* − 1/*p* = *δ*,

(59) r1/pMp(r,h(ν)) ≤21/p(1−r)−δ−1∫r1(1−s)δMp(s,g(ν))ds,

which was to be proved.

**Remark 24**

Before going further note that an immediate consequence of this lemma is the validity of implication (b)⇒(a) of Theorem 11 in the case *X* = *H*_*α*,*ν*_^*p*^ = *H*_*α*,*ν*_^*p*,*∞*^. Then we use this fact and the following two ones to show that *ℒ* maps *h*_*α*,*ν*_^*p*^ into *h*_*α*,*ν*_^*p*^. The set *𝒫* (all polynomials) is dense in *h*_*α*,*ν*_^*p*^ and *ℒ* maps *𝒫* into *𝒫*.

**Lemma 25**

If 0 < *q* < *∞* and *ℒg* is well defined, then

(60) rq/pMpq(r,(ℒg)(ν)) ≤C(1−r)−(δ+1−ɛ)q  ×∫r1Mpq(s,g(ν))(1−s)(δ+1−ɛ)q−1ds,

where

(61) $$\begin{array}{c} ɛ=\{\begin{array}{cc} =0, & q\leq 1,\\ >0, & q>1, \end{array} \end{array}$$

and *C* is independent of *g* and *r*.

In the case *q* ≤ 1, this lemma follows from Lemma 22 and the following.

Sublemma 25.1.* Let *0 < *q* < 1, *β* = 1 + *δ* > 0*, and let u* : (0,1)↦[0, *∞*)* be a nondecreasing function. Then*

(62) (∫r1u(s)(1−s)β−1  ds)q≤C∫r1u(s)q(1−s)qβ−1ds,

*where C is independent of u*.

**Proof**

Fix *r* ∈ (0,1), and define the function *v* on (0,1) by

(63) $$\begin{array}{c} v\left(s\right)=\{\begin{array}{cc} 0, & 0<s\leq r,\\ u\left(s\right), & r<s<1. \end{array} \end{array}$$

Then the desired inequality can be written as

(64) $$\begin{array}{c} {\left(\int_{0}^{1}v\left(s\right){\left(1-s\right)}^{\beta -1}ds\right)}^{q}\leq C\int_{0}^{1}v{\left(s\right)}^{q}{\left(1-s\right)}^{\beta q-1}ds. \end{array}$$

Let *r*_*n*_ = 1 − 2^−*n*^, where  *n* is a nonnegative integer. Then

(65) (∫01v(s)(1−s)β−1ds)q =(∑n=0∞∫rnrn+1v(s)(1−s)β−1ds)q ≤C(∑n=0∞v(rn+1)2−nβ)q ≤C∑n=0∞(v(rn+1)2−nβ)q.

In a similar way we can prove that

(66) ∫01v(s)q(1−s)βq−1ds≥c∑n=0∞v(rn)q2−nβq=c1∑n=−1∞v(rn+1)q2−nβq≥c1∑n=0∞v(rn+1)q2−nβq,

where *c*_1_ = const. >0. Comparing these inequalities we get the result.

In the case *q* > 1, Lemma 25 is a consequence of the following fact.

Sublemma 25.2.* Let *1 < *q* < *∞*,  *ɛ* > 0*, and u* ≥ 0*, a measurable function defined on *(*r*, 1).* Then*

(67) (∫r1u(s)(1−s)δds)q ≤C(1−r)ɛq∫r1u(s)q(1−s)(1+δ−ɛ)q−1ds,

*where C is independent of u*.

**Proof**

Let *κ* = *δ* + 1 − *ɛ* − 1/*q*. Then, by Hölder's inequality (1/*q* + 1/*q*′ = 1),

(68) ∫r1u(s)(1−s)δds =∫r1u(s)(1−s)γ(1−s)δ−γds ≤(∫r1u(s)q(1−s)γq  ds)1/q(∫r1(1−s)(δ−γ)q′  ds)1/q′.

Since (*δ* − *γ*)*q*′ = (*ɛ* − 1 + 1/*q*)*q*′ > −1 because *ɛ* > 0, we have that the last integral is less than

(69) $$\begin{array}{c} C{\left(1-r\right)}^{\delta -\gamma +1/q^{\prime }}=C{\left(1-r\right)}^{ɛ}. \end{array}$$

The result follows.

**Proof of Theorem 11, ((b)⇒(a))**

By Remark 24, we may assume that *q* is finite, which implies that *α* > 0. Let *q* > 1. A standard application of the maximum modulus principle for analytic functions gives

(70) ∫01Mpq(r,(ℒg)(ν))(1−r)qα−1dr ≤C∫01rq/pMpq(r,(ℒg)(ν))(1−r)qα−1dr.

Then, if *q* > 1, we have

(71) ∫01Mpq(r,(ℒg)(ν))(1−r)qα−1dr ≤C∫01(1−r)(α−δ−1+ɛ)q−1dr   ×∫r1Mpq(s,g)(1−s)(δ+1−ɛ)q−1ds =C∫01Mpq(s,g)(1−s)(δ+1−ɛ)q−1ds   ×∫0s(1−r)(α−δ−1+ɛ)q−1dr.

Since *α* − *δ* − 1 = *α* + 1/*p* − *ν* − 1 < 0, we can choose *ɛ* > 0 so that *α* − *δ* − 1 + *ɛ* < 0. Then

(72) $$\begin{array}{c} \int_{0}^{s}{\left(1-r\right)}^{(\alpha -\delta -1+ɛ)q-1}dr\leq C{\left(1-s\right)}^{(\alpha -\delta -1+ɛ)q}. \end{array}$$

Combining this with the preceding inequality we get the result in the case *q* > 1. In the case *q* ≤ 1 the proof is similar and simpler and is omitted.

**Proof of Theorem 11 ((a)⇒(b))**

As noted in Section 2, it is enough to prove Propositions 13 and 15.

**Proof of Proposition 13**

We have

*Case (1).* By Proposition 9 we have *h*_*β*,*ν*_^*∞*^ ⊂ *h*_*α*,*ν*_^*p*^, where *β* = *α* + 1/*p*. Since *κ*_*∞*,*β*,*ν*_ = *κ*_*α*,*p*,*ν*_, it is enough to consider the case of *h*_*β*,*ν*_^*∞*^. Let *f*_*ρ*_(*z*) = 1/(1 − *ρz*),  0 < *ρ* < 1. It is clear that $f_{\rho }\in H(\bar{𝔻})$. A simple calculation shows that the set {*f*_*ρ*_ : 0 < *ρ* < 1} is bounded in *h*_*α*,*ν*_^*∞*^. Hence, if $\bar{ℒ}$ has an extension to a bounded operator from *h*_*α*,*ν*_^*∞*^ to *H*(*𝔻*), then the set $\left\{\bar{ℒ}f_{r}(0)\right\}$ is bounded because the functional *g* ↦ *g*(0) is bounded on *H*(*𝔻*). However,

(73) $$\begin{array}{c} \bar{ℒ}f_{r}\left(0\right)=\sum_{n=0}^{\infty }\frac{r^{n}}{n+1}⟶\infty ,\;\;r⟶1^{-}, \end{array}$$

which is a contradiction. This proves the desired result in the case of *h*_*α*,*ν*_^*p*^. Since *h*_*α*,*ν*_^*p*^ ⊂ *H*_*α*,*ν*_^*p*^, we see that the result holds for this space as well.

*Case (2).* Let *k*_*p*,*α*,*ν*_ < 0 and choose *β* so that *k*_*p*,*β*,*ν*_ = 0. This implies that *β* < *α*. Then it is easy to check that *H*_*α*,*ν*_^*p*,*q*^ ⊂ *H*_*α*,*ν*_^*p*^, which together with the Case (1) gives the result.

**Proof of Proposition 15**

The proof of Proposition 15 is more delicate. First note that, by Proposition 9,

(74) $$\begin{array}{c} H_{\beta ,\nu }^{1,q}\subset H_{\alpha ,\nu }^{p,q}\subset H_{\gamma ,\nu }^{\infty ,q}, \end{array}$$

where

(75) $$\begin{array}{c} \alpha =\beta +1-\frac{1}{p},\;\;\gamma =\alpha +\frac{1}{p}, \end{array}$$

and that

(76) $$\begin{array}{c} \kappa_{1,\gamma ,\nu }=\kappa_{p,\alpha ,\nu }=\kappa_{\infty ,\beta ,\nu }=0, \end{array}$$

see (34). Therefore, it is enough to prove that the function *f* defined by (48) belongs to *H*_*β*,*ν*_^1,*q*^ while *ℒf* does not belong to *H*_*γ*,*ν*_^*∞*,*q*^.

It is easier to prove that *ℒf* ∉ *H*_*γ*,*ν*_^*∞*,*q*^. Namely, since the coefficients *c*_*n*_ of (*ℒf*)^(*ν*)^ are nonnegative, we see that *ℒf* is in *H*_*γ*,*ν*_^*∞*,*q*^ if and only if

(77) $$\begin{array}{c} \int_{0}^{1}{\left(\sum_{n=0}^{\infty }c_{n}r^{n}\right)}^{q}{\left(1-r\right)}^{q\gamma -1}dr<\infty , \end{array}$$

which, by a theorem of *L* ^*p*^-integrability of power series with positive coefficients (see [23, Theorem  1]), is equivalent to

(78) $$\begin{array}{c} \sum_{n=0}^{\infty }2^{-nq\gamma }{\left(\sum_{k\in I_{n}}c_{k}\right)}^{q}<\infty . \end{array}$$

Here

(79) $$\begin{array}{c} I_{n}=\left\{k:2^{n-1}\leq k\leq 2^{n+1}-1\right\}\;\left(\text{for}\;n\geq 1\right),\;\;I_{0}=\left\{0\right\}. \end{array}$$

Since *c*_*k*_≍(*k* + 1)^*ν*^ (*k* → *∞*), the latter is equivalent to

(80) $$\begin{array}{c} \sum_{n=0}^{\infty }2^{nq(\nu -\gamma )}{\left(\sum_{k\in I_{n}}b_{k}\right)}^{q}, \end{array}$$

where *b*_*n*_ are coefficients of *ℒf*. Since *b*_*n*_ ↓ 0, we get the equivalent condition

(81) $$\begin{array}{c} \sum_{n=0}^{\infty }2^{nq(\nu -\gamma +1)}b_{2^{n}}^{q}<\infty . \end{array}$$

This condition is not satisfied because *γ* = *ν* + 1 and

(82) $$\begin{array}{c} b_{2^{n}}=\sum_{k=2^{n}}\frac{1}{\left(k+1\right)\log^{ɛ}\left(k+2\right)}≍{\left(n+1\right)}^{ɛ-1}, \end{array}$$

and hence

(83) $$\begin{array}{c} \sum_{n=0}^{\infty }{\left(n+1\right)}^{(ɛ-1)q}=\infty \;\left(\text{because}\;\;\left(ɛ-1\right)q\leq 1\right), \end{array}$$

where the function *ℒf* is well defined because *ɛ* > 1, which implies

(84) $$\begin{array}{c} \sum_{n=0}^{\infty }\frac{1}{\left(n+1\right)\log^{ɛ}\left(n+2\right)}<\infty . \end{array}$$

In proving that *f* ∈ *H*_*β*,*ν*_^1,*q*^ we use a sequence {*V*_*n*_}_0_^*∞*^ constructed in the following way (see, e.g., [24]).

Let *ω* be a *C* ^*∞*^ function on ℝ such that

1. *ω*(*t*) = 1 for *t* ≤ 1,
2. *ω*(*t*) = 0 for *t* ≥ 2,
3. *ω* is decreasing and positive on the interval (1,2).

Let *φ*(*t*) = *ω*(*t*/2) − *ω*(*t*), and let *V*_0_(*z*) = 1 + *z*, and, for *n* ≥ 1,

(85) $$\begin{array}{c} V_{n}\left(t\right)=\sum_{k=0}^{\infty }\varphi \left(\frac{k}{2^{n-1}}\right)z^{k}=\sum_{k=2^{n-1}}^{2^{n+1}}\varphi \left(\frac{k}{2^{n-1}}\right)z^{k}. \end{array}$$

These polynomials have the following properties:

(86) $$\begin{array}{c} g\left(z\right)=\sum_{n=0}^{\infty }V_{n}*g\left(z\right),\;\text{for}\;g\in H\left(𝔻\right); \end{array}$$

(87) $$\begin{array}{c} {||V_{n}*g||}_{p}\leq C{||g||}_{p},\;\text{for}\;g\in H^{p},\;\;p>0; \end{array}$$

(88) $$\begin{array}{c} {||V_{n}||}_{p}≍2^{n(1-1/p)},\;\forall p>0. \end{array}$$

(Here ∗ denotes the Hadamard product).

In [25, Lemma 2.1], the following characterization of *H*_*α*,*ν*_^*p*,*q*^ was proved.

Lemma A. *Let *0 < *p* ≤ *∞, and *0 < *q* ≤ *∞*, *α* > 0,* and let ν be a nonnegative integer. A function g* ∈ *H*(*𝔻*)* is in H*_*α*,*ν*_^*p*,*q*^ *if and only if*

(89) $$\begin{array}{c} K_{1}\left(g\right):={\left(\sum_{n=0}^{\infty }2^{n(\nu -\alpha )q}{||V_{n}*g||}_{p}^{q}\right)}^{1/q}<\infty , \end{array}$$

*and we have K*_1_(*g*)≍||*f*||_*H*_*α*,*ν*_^*p*,*q*^_.* In the case of H*_*α*,*ν*_^*p*^ *(resp., h*_*α*,*ν*_^*p*^ *) this is interpreted as *||*V*_*n*_∗*g*||_*p*_ = *𝒪*(2^*n*(*α*−*ν*)^)* (resp. *||*V*_*n*_∗*g*||_*p*_ = *o*(2^*n*(*α*−*ν*)^)*).*

**Remark 26**

This lemma was deduced in [25] from the case *ν* = 0 (which is relatively easy to discuss) by using some nontrivial results of Hardy and Littlewood [26] and of Flett (see [27]). By a successive application of Lemma 27 below (case *δ* = 0), we can make this deduction elementary.

**Lemma 27**

If *n* is a positive integer,

(90) $$\begin{array}{c} P\left(z\right)=\sum_{k=n}^{4n}\lambda_{k}z^{k}, \end{array}$$

where {*λ*_*k*_} is a complex sequence, and

(91) $$\begin{array}{c} Q\left(z\right)=\sum_{k=n}^{4n}{\left(k+1\right)}^{\beta }\log^{\delta }\left(k+1\right)\lambda_{k}z^{k},\;\delta ,\beta \in \mathbb{R}, \end{array}$$

then there is a constant *C* depending only on *δ* and *β* such that

(92) $$\begin{array}{c} C^{-1}{||Q||}_{1}\leq {\left(n+1\right)}^{\beta }\log^{\delta }\left(n+1\right){||P||}_{1}\leq C{||Q||}_{1}. \end{array}$$

**Proof**

The proof can be reduced to two cases: (1) *β* = 0, *δ* ∈ ℝ and (2) *δ* = 0, *β* ∈ ℝ. We will consider only Case (1); Case (2) is discussed similarly.

Let *ψ* be a *C* ^*∞*^ function on (0, *∞*) such that supp⁡*ψ* ⊂ (1/2,5) and *ψ*(*t*) = 1 for 1 ≤ *t* ≤ 4. Then we have *Q* as

(93) $$\begin{array}{c} Q\left(z\right)=\sum_{k=0}^{\infty }\psi \left(\frac{k}{n}\right)\log^{\delta }\left(k+2\right)\lambda_{k}z^{k}, \end{array}$$

where *λ*_*k*_ : = 0 for *k* ∉ [*n*, 2*n*]. Fix *n* and let

(94) $$\begin{array}{c} \Delta^{2}\eta \left(k\right)=\eta \left(k\right)-2\eta \left(k+1\right)+\eta \left(k+2\right), \end{array}$$

where

(95) $$\begin{array}{c} \eta \left(t\right)=\psi \left(\frac{t}{n}\right)\log^{\delta }\left(t+1\right). \end{array}$$

We have

(96) Q(z)=∑k=0∞Δ2η(k)(k+1)(σkP)(z) =∑n/2≤k≤5nΔ2η(k)(k+1)(σkP)(z),

where *σ*_*k*_ *P* are (*C*, 1)-means of *P*. It follows that

(97) ||Q||1≤∑n/2≤k≤5n|Δ2η(k)|(k+1)||σkP||1≤∑n/2≤k≤5n|Δ2η(k)|(k+1)||P||1,

where we have used Fejér's inequality ||*σ*_*k*_ *P*||_1_ ≤ ||*P*||_1_. Now we use Lagrange's inequality in the form

(98) $$\begin{array}{c} \left|\Delta^{2}\eta \left(k\right)\right|\leq 2\underset{k\leq t\leq k+2}{\sup}\left|\eta^{\prime \prime }\left(t\right)\right| \end{array}$$

and the easily proved inequality

(99) $$\begin{array}{c} \left|\eta^{\prime \prime }\left(t\right)\right|\leq \frac{C}{n^{2}}\log^{\delta }\left(n+1\right),\;\frac{n}{2}\leq t\leq 5n \end{array}$$

to get

(100) ||Q||1≤Cn2log⁡δ(n+1)∑n/2≤k≤5n(k+1)||P||1≤Clog⁡δ(n+1)||P||1,

which proves the desired result in one direction. To prove the reverse inequality we write *P* as

(101) $$\begin{array}{c} P\left(z\right)=\sum_{k=n}^{4n}\log^{-\delta }\left(k+1\right)\xi_{k}z^{k},\;\text{where}\;\;\xi_{k}=\lambda_{k}\log^{\delta }\left(k+1\right). \end{array}$$

Applying the above case, we get

(102) ||P||1≤Clog⁡−δ(n+1)||Q||1,

which completes the proof.

**Proof of Proposition 15**

As noted above it is enough to prove that *f* ∈ *H*_*β*,*ν*_^1,*q*^, where *β* = *ν*. In this case, by Lemma A, the function *f* belongs to *H*_*β*,*ν*_^1,*q*^ if and only if

(103) $$\begin{array}{c} \sum_{n=0}^{\infty }{||V_{n}*f||}_{1}^{q}<\infty . \end{array}$$

On the other hand, by Lemma 27 and the property (88),

(104) $$\begin{array}{c} {||V_{n}*f||}_{1}^{q}≍{\left(\log^{ɛ}\left(2^{n+1}\right){||V_{n}||}_{1}\right)}^{q}≍{\left(n+1\right)}^{-ɛq}, \end{array}$$

which implies that *f* is in *H*_*β*,*ν*_^1,*q*^, because *ɛq* > 1.

Thus, we have proved the implication (a)⇒(b) of Theorem 11.

## 4. Proof of Theorem 16

It is clear that (b) implies (a). We have already noted that (c) implies (b). The following assertion shows that (d) implies (c).

**Proposition**

If *q* ≤ 1,  1 ≤ *p* ≤ 2,  *κ*_*p*,*α*,*ν*_ = *ν* + 1 − *α* − 1/*p* = 0, and *g* ∈ *H*_*α*,*ν*_^*p*,*q*^, then

(105) $$\begin{array}{c} \sum_{n=0}^{\infty }\frac{\left|\hat{g}\left(n\right)\right|}{n+1}<\infty . \end{array}$$

**Proof**

Since *H*_*α*,*ν*_^1,*q*^ ⊂ *H*_*α*,*ν*_^1,1^, it is enough to consider the case *q* = 1. Let

(106) $$\begin{array}{c} P_{n}=V_{n-1}+V_{n}+V_{n+1},\;\;n\geq 0,\;\;\text{where}\;\;V_{-1}=0. \end{array}$$

Since *P*_*n*_∗*V*_*n*_ = *V*_*n*_, which follows from (86) and the fact *V*_*n*_∗*V*_*m*_ = 0 for |*m* − *n* | ≥2, we see that

(107) $$\begin{array}{c} \text{Lemma}\;\;\text{A}\;\;\text{remains}\;\;\text{true}\;\;\text{if}\;\;V_{n}\;\;\text{is}\;\;\text{replaced}\;\;\text{by}\;\;P_{n}. \end{array}$$

Also the relation *P*_*n*_∗*V*_*n*_ = *V*_*n*_ implies that ${\hat{P}}_{n}(k){\hat{V}}_{n}(k)={\hat{V}}_{n}(k)$, and hence ${\hat{P}}_{n}(k)=1$ whenever ${\hat{V}}_{n}(k)\neq 0$. In particular ${\hat{P}}_{n}(k)=1$ for *k* ∈ *I*_*n*_ (see (79)). We use Hardy's inequality

(108) $$\begin{array}{c} \sum_{k=0}^{\infty }{\left(k+1\right)}^{p-2}{\left|\hat{h}\left(k\right)\right|}^{p}\leq C{||h||}_{p}^{p},\;1\leq p\leq 2, \end{array}$$

and Lemma (107) to conclude that if *g* ∈ *H*_*α*,*ν*_^*p*,1^, then

(109) $$\begin{array}{c} \sum_{n=0}^{\infty }2^{-n/p}{\left(\sum_{k\in I_{n}}{\left|\hat{g}\left(k\right)\right|}^{p}\right)}^{1/p}<\infty . \end{array}$$

Hence, by Hölder's inequality,

(110) ∑n=0∞2−n∑k∈In|g^(k)| ≤∑n=0∞2−n(∑k∈In|g^(k)|p)1/p2n(1−1/p) =∑n=0∞2−n/p(∑k∈In|g^(k)|p)1/p,

which proves the result.

It remains to be proven that (a) implies (d); that is, that (a) does not hold in the following cases:

1. 1 ≤ *p* ≤ *∞*,  0 < *q* ≤ *∞*, and *κ*_*p*,*α*,*ν*_ < 0;
2. 1 ≤ *p* ≤ 2, 1 < *q* ≤ *∞*, *κ*_*p*,*α*,*ν*_ = 0;
3. 2 < *p* ≤ *∞*, 0 < *q* ≤ *∞*, *κ*_*p*,*α*,*ν*_ = 0.

Case (1) is part of Proposition 13. In view of the same proposition, we can assume, in what follows, that *q* < *∞*.

The following assertion proves the desired result in Cases (2) and (3, *q* > 1).

**Proposition**

If 1 ≤ *p* ≤ *∞*, *κ*_*p*,*α*,*ν*_ = 0, and 1 < *q* < *∞*, then $\bar{ℒ}$ cannot be extended to a bounded operator from *H*_*α*,*ν*_^*p*,*q*^ to *H*(*𝔻*).

**Proof**

(a) Let

(111) fɛ,ρ(z)=∑k=0∞ρnzn(k+1)log⁡1+ɛ(k+2),          ɛ>0,  0<ρ≤1.

It follows from Lemmas A and 27 that

(112) ||fɛ,1||α,νp,qq  ≍∑n=0∞(n+1)−(1+ɛ)q≤∑n=0∞(n+1)−q=Cq<∞,

where *C*_*q*_ is independent of *ɛ*. This inequality remains true if *f*_*ɛ*,1_ is replaced by *f*_*ɛ*,*ρ*_ with *ρ* < 1. The function *f*_*ɛ*,*ρ*_(*ρ* < 1) belongs to $H(\bar{𝔻})$ and the set {*f*_*ɛ*,*ρ*_ : *ɛ* > 0, *ρ* < 1} is bounded in *H*_*α*,*ν*_^*p*,*q*^. On the the other hand,

(113) ℒfɛ,ρ(0)=∑k=0∞ρn(k+1)log⁡1+ɛ(k+2)⟶∞,             ɛ⟶0+,  ρ⟶1−.

The result follows.

The case when *q* ≤ 1 and *p* > 2 is more delicate and depends on Khinchin's inequality and a deep result of Khinchin and Kolmogorov ([28]; see [14, Ch. V, Sec. 8]).

Theorem H.   *Let R*_*n*_ *denote the sequence of Rademacher functions, R*_*n*_(*t*) = sign⁡(sin(2^*n*^ *πt*)), *n* ≥ 0, 0 ≤ *t* ≤ 1*. If *{*c*_*n*_}* is a sequence in ℂ such that *∑_*n*=0_^*∞*^ | *c*_*n*_|^2^ = *∞, then the series *∑_*n*=0_^*∞*^ *c*_*n*_ *R*_*n*_(*t*)* diverges for almost all t* ∈ [0,1]* and moreover the sequence of its partial sums is unbounded a.e.*

We also need the following theorem of Khinchin [14, Ch. V, Theorem (8.4)].

Theorem I.   *Let *{*c*_*k*_}* be a finite sequence, and let *0 < *q* < *∞. Then*

(114) $$\begin{array}{c} \int_{0}^{1}{\left|\sum_{k}c_{k}R_{k}\left(t\right)\right|}^{q}≍{\left(\sum_{k}{\left|c_{k}\right|}^{2}\right)}^{q/2}, \end{array}$$

*where the “involving” constants depend only on q*.

Let

(115) $$\begin{array}{c} \Delta_{n}\left(z\right)=\sum_{k\in I_{n}}z^{k},\;\;\Delta_{n}g=\Delta_{n}*g. \end{array}$$

The following fact was proved in [29].

Lemma B.   *Let *1 < *p* < *∞*, 0 < *q* ≤ *∞, and* *α* > 0*. A function g* ∈ *H*(*𝔻*)* is in H*_*α*_^*p*,*q*^ *if and only if*

$$\begin{array}{cc} \left(†\right) & K\left(g\right):={\left(\sum_{n=0}^{\infty }2^{-n\alpha q}{||\Delta_{n}g||}_{p}^{q}\right)}^{1/q}<\infty , \end{array}$$

*and we have K*(*g*)≍||*f*||__*α*_^*p*,*q*^_ *. In the case of H*_*α*_^*∞*^ *(resp., h*_*α*_^*p*^ = *h*_*α*,0_^*p*^ *), relation *((†))* means *||Δ_*n*_ *g*||_*p*_ = *𝒪*(2^*nα*^)* (resp., *||Δ_*n*_ *g*|| = *o*(2^*nα*^)*, as n* → *∞).*

As a consequence of this lemma and Lemma 27 we have the following.

**Lemma 30**

Let 1 < *p* < *∞*, 0 < *q* ≤ *∞*, and *α* > 0. A function *g* ∈ *H*(*𝔻*) is in *H*_*α*,*ν*_^*p*,*q*^ if and only if

$$\begin{array}{cc} \left(‡\right) & K_{\nu }\left(g\right):={\left(\sum_{n=0}^{\infty }2^{n(\nu -\alpha )q}{||\Delta_{n}g||}_{p}^{q}\right)}^{1/q}<\infty , \end{array}$$

and we have *K*_*ν*_(*g*)≍||*f*||__*α*,*ν*_^*p*,*q*^_. In the case of *H*_*α*,*ν*_^*p*^, respectively, *h*_*α*,*ν*_^*p*^, relation ((‡)) is interpreted as ||Δ_*n*_ *g*||_*p*_ = *𝒪*(2^(*α*−*ν*)*n*^), respectively, ||Δ_*n*_ *g*||_*p*_ = *o*(2^(*α*−*ν*)*n*^).

**Lemma 31**

If 0 < *p* < *∞* and 0 < *q* < *∞*, and *g*_*t*_(*z*) = ∑_*k*=0_^*∞*^ *c*_*k*_ *z* ^*k*^ *R*_*k*_(*t*), then

(116) $$\begin{array}{c} \int_{0}^{1}{||\Delta_{n}g_{t}||}_{p}^{q}≍{\left(\sum_{k\in I_{n}}{\left|c_{k}\right|}^{2}\right)}^{q/2},\;n\geq 0. \end{array}$$

**Proof**

In the case *p* = *q*, the relation immediately follows from Theorem I. Let *p* > *q*. Then, by Jensen's inequality for the convex function *x* ↦ *x* ^*p*/*q*^,

(117) (∫01||Δngt||pqdt)p/q≤∫01(||Δngt||pq)p/q=∫01||Δngt||ppdt≍(∑k∈In|ck|2)p/2.

On the other hand, since ||Δ_*n*_ *g*||_*p*_ ≥ ||Δ_*n*_ *g*||_*q*_, we have

(118) ∫01||Δngt||pqdt≥∫01||Δngt||qqdt≍(∑k∈In|ck|2)q/2.

This proves the result in the case *p* > *q*. The remaining case is discussed similarly.

**Proposition 32**

If 0 < *q* ≤ 1,  2 < *p* ≤ *∞*,  *α* > 0, and *κ*_*p*,*α*,*ν*_ = 0, then $\bar{ℒ}$ cannot be extended to a bounded operator from *H*_*α*,*ν*_^*p*,*q*^ to *H*(*𝔻*).

**Proof**

Consider first the case 2 < *p* < *∞*. Let

(119) gt(z)=∑k=0∞(k+1)1/2−1/plog⁡−2/q(k+2)Rn(t)zk,                     0≤t≤1.

Since

(120) $$\begin{array}{c} \sum_{k=0}^{\infty }{\left({\left(k+1\right)}^{1/2-1/p}\log^{-2/q}\left(k+2\right)\right)}^{2}=\infty , \end{array}$$

because 2/*p* < 1, the sequence of partial sums of the series *g*_*t*_(1) diverges on a set *E* ⊂ [0,1] such that |*E* | = 1, which follows from Theorem H. (We can assume that *E* does not contain points where *R*_*n*_(*t*) = 0 because the set of such points is denumerable). On the other hand, by Lemma 31, we have

(121) ∫E||gt||  α,νp,qq≍∫E∑n=0∞2n(1/p−1)q||Δngt||pq≍∑n=0∞2n(1/p−1)q||Δngt||2q≍∑n=0∞2n(1/p−1)q2n(1/2−1/p)q2nq/2(n+1)−2=∑n=0∞(n+1)−2<∞.

It follows that *g*_*τ*_ ∈ *H*_*α*,*ν*_^*p*,*q*^ for at least one *τ* ∈ *E*.

To complete the proof we consider the polynomials ($\in H(\bar{𝔻})$)

(122) $$\begin{array}{c} s_{n}\left(z\right)=\sum_{k=0}^{2^{n+1}-1}{\hat{g}}_{\tau }\left(k\right)z^{k}. \end{array}$$

It follows from Lemma B that ||*s*_*n*_|| ≤ *C*||*g*_*τ*_|| < *∞* (in the norm of *H*_*α*,*ν*_^*p*,*q*^), where *C* is independent of *n*. On the other hand, as noted before, the sequence

(123) $$\begin{array}{c} ℒs_{n}\left(0\right)=\sum_{k=0}^{2^{n+1}-1}{\left(k+1\right)}^{1/2-1/p}\log^{-2}\left(k+2\right)R_{n}\left(\tau \right) \end{array}$$

is not bounded, which proves the result in the case *p* < *∞*.

In the case of *H*_*α*,*ν*_^*∞*,*q*^ we have *ν* + 1 − *α* = 0. Hence, if *g* ∈ *H*_*β*,*ν*_^*p*,*q*^ and *κ*_*p*,*β*,*ν*_ = 0, that is, *ν* − *β* = 1/*p* − 1, then it follows from Proposition 9 that *H*_*β*,*ν*_^*p*,*q*^ ⊂ *H*_*α*,*ν*_^*∞*,*q*^, continuously. The desired result follows from the case *p* < *∞*.

**Remark 33**

If *q* = *∞*, *p* > 2, *ν* = 0, and *κ* = 0, that is, *α* = 1 − 1/*p*, then we can take

(124) $$\begin{array}{c} g_{t}\left(z\right)=\sum_{k=0}^{\infty }{\left(k+1\right)}^{1/2-1/p}R_{n}\left(t\right), \end{array}$$

and apply the above approach to show that there is a function *g* ∈ *H*_*α*_^*p*,*∞*^ such that

$$\begin{array}{cc} \left(**\right) & \left|\hat{g}\left(k\right)\right|≍{\left(k+1\right)}^{1/2-1/p}, \end{array}$$

and hence

(125) $$\begin{array}{c} \underset{n\to \infty }{\lim}\frac{\hat{g}\left(n\right)}{n+1}=\infty . \end{array}$$

It is not easy to give a concrete example of such a function. A “natural” example is

(126) $$\begin{array}{c} g\left(z\right)={\left(1-z\right)}^{1/p-3/2}, \end{array}$$

whose coefficients satisfy ((∗∗)). However,

(127) $$\begin{array}{c} \int_{0}^{2\pi }{\left|1-re^{i\theta }\right|}^{p(1/p-3/2)}d\theta ≍{\left(1-r\right)}^{1-3p/2}. \end{array}$$

If *g* ∈ *H*_*α*_^*p*,*∞*^, then

(128) $$\begin{array}{c} {\left(1-r\right)}^{1-3p/2}\leq C{\left(1-r\right)}^{p(1/p-1)}, \end{array}$$

which implies *p*(1/*p* − 1) ≤ 1 − 3*p*/2, while this implies *p* ≤ 2, a contradiction which shows that *g* ∉ *H*_*α*_^*p*,*∞*^, for *p* > 2.

## 5. Proof of Theorem 18

For technical reasons, we introduce the space

(129) $$\begin{array}{c} \ell_{-1}^{1}=\left\{g\in H\left(𝔻\right):\sum_{n=0}^{\infty }\frac{\left|\hat{g}\left(n\right)\right|}{n+1}<\infty \right\}. \end{array}$$

In the proof of Proposition 34 we use the following deep result of Kisliakov [30].

Theorem J.* For any sequence *{*c*_*k*_}_*k*=*m*_^*n*^ *there is a polynomial h*(*z*) = ∑_*k*=*m*_^*n*^ *b*_*k*_ *z* ^*k*^ *such that *|*b*_*k*_ | ≥|*c*_*k*_|* and*

(130) $$\begin{array}{c} {||h||}_{\infty }\leq C{\left(\sum_{k=m}^{n}{\left|c_{k}\right|}^{2}\right)}^{1/2}, \end{array}$$

*where C is an absolute constant.*

**Proposition 34**

Let *κ*_*p*,*α*,*ν*_ > 0. If (1) 1 < *q* ≤ *∞*, 2 < *p* ≤ *∞*, and *ν* − *α* ≤ −1/2, or (2) 0 < *q* ≤ 1, 2 < *p* ≤ *∞*, and *ν* − *α* < −1/2, then *H*_*ν*,*α*_^*p*,*q*^⊈*ℓ*_−1_^1^.

**Proof**

We have

*Case (1),*  (*p* < *∞*). Let *q* = *∞* and let

(131) $$\begin{array}{c} g_{t}\left(z\right)=\sum_{k=0}^{\infty }c_{k}R_{k}\left(t\right)z^{k}, \end{array}$$

where

(132) $$\begin{array}{c} {\left(\sum_{k\in I_{n}}{\left|c_{k}\right|}^{2}\right)}^{1/2}\leq C2^{n(\alpha -\nu )}. \end{array}$$

Take *c*_*k*_ = (*k* + 1)^*ξ*^. Then

(133) $$\begin{array}{c} {\left(\sum_{k\in I_{n}}c_{k}^{2}\right)}^{1/2}≍2^{n\xi }2^{n/2}\leq C2^{n(\alpha -\nu )}, \end{array}$$

whence we can choose *ξ* + 1/2 = *α* − *ν*; that is, *ξ* = *α* − *ν* − 1/2 ≥ 0.

We have, by Lemma 31,

(134) ∫01||Δnft||p≤C(∑k∈Inck2)1/2≤C2n(α−ν).

This implies that there is a sequence *ε*_*n*_ ∈ {−1,1} such that the function

(135) $$\begin{array}{c} h\left(z\right)=\sum_{k=0}^{\infty }c_{k}\varepsilon_{k}z^{k} \end{array}$$

belongs to *H*_*α*,*ν*_^*p*,*∞*^. On the other hand,

(136) $$\begin{array}{c} \sum_{k=0}^{\infty }\frac{\left|\hat{h}\left(k\right)\right|}{k+1}=\infty . \end{array}$$

If 1 < *q* < *∞*, we consider the function

(137) $$\begin{array}{c} g_{t}\left(z\right)=\sum_{n=0}^{\infty }c_{n}R_{n}\left(t\right)z^{k},\;\text{where}\;c_{n}=\frac{{\left(n+1\right)}^{\xi }}{\log\left(n+2\right)}, \end{array}$$

and proceed as above to get the result.

*Case (1),*  (*p* = *∞*). Choose {*c*_*k*_} as above and consider the function *h*(*z*) = ∑_*k*=0_^*∞*^ *b*_*k*_ *z* ^*k*^, where |*b*_*k*_ | ≥*c*_*k*_ and ||Δ_*n*_ *h*||_*∞*_ ≤ *C*(∑_*k*∈*I*_*n*__ *c*_*k*_^2^)^1/2^ (Theorem J, Kisliakov). Finally we use the inequality

(138) $$\begin{array}{c} {||h||}_{\alpha ,\nu }^{p,q}\leq C{\left(\sum_{k\in I_{n}}2^{(\nu -\alpha )q}{||\Delta_{n}h||}_{\infty }^{q}\right)}^{1/q}, \end{array}$$

(see [29, Theorem 2.1(a)]) to finish the proof of Case (1).

In Case (2) we choose $\hat{g}(n)={\left(n+1\right)}^{\xi }$, where 0 < *ξ* < *α* − *ν* − 1/2, and repeat the above reasoning to complete the proof.

**Proposition 35**

If *ν* − *α* = −1/2, 0 < *q* ≤ 1, and 2 < *p* ≤ *∞*, then *H*_*α*,*ν*_^*p*,*q*^ ⊂ *ℓ*_−1_^1^.

**Proof**

We have

(139) ∑n=0∞2n(ν−α)q||Δng||pq≥∑n=0∞2n(ν−α)q||Δng||2q≥(∑n=0∞2n(ν−α)||Δng||2)q=(∑n=0∞2−n/2||Δng||2)q≥(∑n=0∞2−n∑k∈In|g^(k)|)q,

which completes the proof of the proposition.

**Proposition 36**

If 2 < *p* ≤ *∞*, *κ*_*p*,*α*,*ν*_ > 0, 0 < *q* ≤ *∞*, and *ν* − *α* > −1/2, then *H*_*α*,*ν*_^*p*,*q*^ ⊂ *ℓ*_−1_^1^.

**Proof**

Let *g* ∈ *H*_*α*,*ν*_^*p*,*q*^. Then *g* ∈ *H*_*α*,*ν*_^*p*,*∞*^, and hence *g* ∈ *H*_*α*,*ν*_^2,*∞*^. It follows that ||Δ_*n*_ *g*||_2_ ≤ *c*2^*n*(*α*−*ν*)^. On the other hand,

(140) ∑n=0∞2−n∑k∈In|g^(k)|≤C∑n=0∞2−n/2||Δng||2≤C∑n=0∞2−n/22n(α−ν)<∞,

because −1/2 + *α* − *ν* < 0. This proves the proposition.

The following proposition completes the proof of Theorem 18.

**Proposition**

If 1 ≤ *p* ≤ 2, *κ*_*p*,*α*,*ν*_ > 0, and 0 < *q* ≤ *∞*, then *H*_*α*,*ν*_^*p*,*q*^ ⊂ *ℓ*_−1_^1^.

**Proof**

Let *g* ∈ *H*_*α*,*ν*_^*p*,*q*^. Choose *β* so that *κ*_*p*,*α*,*ν*_ = *κ*_2,*β*,*ν*_; that is, *α* = *β* + 1/2 − 1/*p*. Then, by Proposition 9(b), we have *H*_*α*,*ν*_^*p*,*q*^ ⊂ *H*_*β*,*ν*_^2,*q*^. This implies that *H*_*α*,*ν*_^*p*,*q*^ ⊂ *H*_*β*,*ν*_^2,*∞*^ which means that ||Δ_*n*_ *g*||_2_ ≤ *C*2^*n*(*β*−*ν*)^, and so

(141) $$\begin{array}{c} 2^{-n/2}{||\Delta_{n}g||}_{2}\leq C2^{n(\beta -\nu -1/2)}. \end{array}$$

It follows that

(142) 2−n∑k∈In|g^(k)|≤C2n(β−ν−1/2)=C2n(α−ν+1/p−1)=C2−nκp,α,ν.

The result follows.

## 6. On the Condition $\left|\hat{g}\left(n\right)\right|\geq {\left(n+1\right)}^{\eta }$, *η* > 0

In this section we suppose that *k*_*p*,*α*,*ν*_ = *ν* − *α* + 1 − 1/*p* > 0; that is, *ℒ* acts as an operator from *H*_*α*,*ν*_^*p*,*q*^ into *H*_*α*,*ν*_^*p*,*q*^(*α* > 0). We want to analyze the proof of Proposition 34 more carefully. Throughout the section we assume that 2 < *p* ≤ *∞* and *κ*_*p*,*α*,*ν*_ > 0, so that *ℒ* maps *H*_*α*,*ν*_^*p*,*q*^ into *H*_*α*,*ν*_^*p*,*q*^ by Theorem 11.

**Proposition**

Let *q* = *∞* and *ν* − *α* ≤ −1/2. Then (i) there exists a function *g* ∈ *H*_*α*,*ν*_^*p*,*q*^ such that $|\hat{g}(n)|\geq {\left(n+1\right)}^{\xi }$, where *ξ* = *α* − *ν* − 1/2 ≥ 0. (ii) The exponent *ξ* is best possible. (iii) If *ξ* > 0, then there is an *η* > 0 and a function *g* ∈ *H*_*α*,*ν*_^*p*,*q*^ such that $|\hat{g}(n)|\geq {\left(n+1\right)}^{\eta }$.

**Proof**

Statement (i) follows from the proof of Proposition 34. (ii) In order to prove that *ξ* is best possible we use Lemma 30, which states that *g* ∈ *H*_*α*,*ν*_^*p*,*∞*^ if and only if ||Δ_*n*_ *g*||_*p*_ ≤ *C*2^*n*(*α*−*ν*)^, which implies that ||Δ_*n*_ *g*||_2_ ≤ *C*2^(*α*−*ν*)*n*^. Assuming that $|\hat{g}(k)|\geq {\left(n+1\right)}^{s}$ we get

(143) $$\begin{array}{c} 2^{ns}2^{n/2}\leq C2^{n(\alpha -\nu )}, \end{array}$$

whence *s* + 1/2 ≤ *α* − *ν*; that is, *s* ≤ *α* − *ν* − 1/2 = *ξ*.

(iii) This follows from (i).

**Proposition 39**

Let 1 < *q* < *∞* and *ν* − *α* ≤ −1/2. Then (i) there exists a function *g* ∈ *H*_*α*,*ν*_^*p*,*q*^ such that $\left|\hat{g}(n)|\geq {\left(n+1\right)}^{\xi }/\log(n+2\right)$. (ii) There is no function *g* ∈ *H*_*α*,*ν*_^*p*,*q*^ such that $|\hat{g}(n)|\geq {\left(n+1\right)}^{\xi }$. (iii) If *ξ* > 0 and 0 < *η* < *ξ*, then there exists a function *g* such that $|\hat{g}(n)|\geq {\left(n+1\right)}^{\eta }$.

**Proof**

Assertion (i) is part of the proof of Proposition 34. To prove (ii) we use the fact that *H*_*α*,*ν*_^*p*,*q*^ ⊂ *h*_*α*,*ν*_^*p*^, which implies that ||Δ_*n*_ *g*||_2_ = *o*(2^*n*(*α*−*ν*)^). If $|\hat{g}(n)|\geq {\left(n+1\right)}^{\xi }$, then the latter implies that

(144) $$\begin{array}{c} 2^{n\xi }2^{n/2}=o\left(2^{n(\alpha -\nu )}\right), \end{array}$$

and hence 1 = *o*(1), which is impossible.

In a similar way one proves the following.

**Proposition**

Let *q* ≤ 1 and *ξ* = *α* − *ν* − 1/2 > 0. Then (i) if 0 < *η* < *ξ*, then there is a function *g* ∈ *H*_*α*,*ν*_^*p*,*q*^ such that $|\hat{g}(n)|\geq {\left(n+1\right)}^{\eta }$. (ii) In (i), *η* cannot be replaced by *ξ*.

Combining the above propositions we get the following.

**Theorem**

Let *ℒ* map *H*_*α*,*ν*_^*p*,*q*^ into itself, and *p* > 2. Then the following statements are equivalent:

- there is a function *g* ∈ *H*_*α*,*ν*_^*p*,*q*^ such that $|\hat{g}(n)|\geq {\left(n+1\right)}^{\eta }$ for some *η* > 0;
- *ν* − *α* < −1/2.

Moreover, if *ν* − *α* < −1/2 and *η* ∈ (0, *α* − *ν* + 1/2) is arbitrary, then there is *g* ∈ *H*_*α*,*ν*_^*p*,*q*^ such that $|\hat{g}(n)|\geq {\left(n+1\right)}^{\eta }$. If in addition *q* = *∞*, we can take *η* = *α* − *ν* − 1/2.
